# Supplementary material for: ‘The Addiction Was Making Things Harder for My Mental Health’: A Qualitative Exploration of the Views of Adults and Adolescents Accessing a Substance Misuse Treatment Service
Source: Int J Environ Res Public Health. 2023 May 26;20(11):5967. doi: 10.3390/ijerph20115967 (PMC10253073; doi:10.3390/ijerph20115967)
Supplement: Supplementary file 1 [file ijerph-20-05967-s001.zip › ijerph-2339089-supplementary.pdf]

Interview Schedule

Introduction

1. Ensure participants understand the aims of the study, and provide consent to participate (including being happy to have the interview audio recorded), do they have any questions?
2. To start I wondered if you could introduce yourself, please?
3. Could we talk a little bit about your life in general now and what brought you here?

Part 1 – Creating a timeline (skip this part for adult participants)

I'd like to draw up a sort of timeline of your life, if that is that OK?

Prompts:

- How old are you now?
- Are you still at school/college?
- When did you start/finish school?
- What about primary school?
- Who do you live with at the moment?
- (If they have siblings) Are they older or younger?
- Have you always lived with the same people?
- Have you ever moved house? (When did you move?)
- Are there any other important things that you think should be on that timeline?

Part 2 – Substance use

Okay so next what I'd like to do is talk a little bit about alcohol and drug use [YP: and if you have drunk alcohol or used drugs see where that fits on the timeline]. Is that OK? Remember if there's anything you don't want to answer just say so and we'll move on.

Prompts:

- Do you drink alcohol?
- (If yes) When did you have your first drink?
- (If no) have you ever had a drink of alcohol?
- Have you ever taken any other drugs?
- Would you mind telling me which drugs you have taken?
- And when did you first start taking…?
- Do you still take…?
- (If still using) Is your drinking/drug use something you want to change?

Part 3 – Experience of treatment

What do you think about the treatment service?

Prompts:

- Could you tell me how you came to be referred to the treatment service?

- Was that the result of a specific event or a general build up over time?
- Were you given an assessment appointment? How did you find out about that? By letter, phone call, text message?
- Was there anything that made you decide to go/not go?
- Was there anything that could have changed your mind?
- Could you tell me a little more about that appointment like what it involved?
- How did you feel going along to that appointment?
- How did you feel talking to staff during that appointment?
- Have you had any more appointments at the treatment centre? Could you tell me a bit about them?
- Was there anything particular that made you decide to go/not go?
- Was there anything that could have changed your mind?
- Do you ever see or talk to anyone else that goes to the treatment centre?
- Could you tell me a little bit about what you think of them, for example do you get on with them?
- Is there anything that you think could be improved about the treatment centre?

Thank you for sharing that with me. Is there anything you'd like to talk about before we finish?

Provide participants with signpost list to sources of support, and voucher for participation.
